# Hypouricemic Effects of *Ganoderma applanatum* in Hyperuricemia Mice through OAT1 and GLUT9

**DOI:** 10.3389/fphar.2017.00996

**Published:** 2018-01-15

**Authors:** Tianqiao Yong, Shaodan Chen, Yizhen Xie, Diling Chen, Jiyan Su, Ou Shuai, Chunwei Jiao, Dan Zuo

**Affiliations:** ^1^State Key Laboratory of Applied Microbiology Southern China, Guangdong Provincial Key Laboratory of Microbial Culture Collection and Application, Guangdong Open Laboratory of Applied Microbiology, Guangdong Institute of Microbiology, Chinese Academy of Sciences, Guangzhou, China; ^2^Guangdong Yuewei Edible Fungi Technology Co., Ltd., Guangzhou, China; ^3^Guangzhou Institutes of Biomedicine and Health, Chinese Academy of Sciences, Guangzhou, China

**Keywords:** *Ganoderma applanatum*, hyperuricemia, uric acid transporter 1, organic anion transporter 1, glucose transporter 9, concentrative nucleoside transporter 2

## Abstract

*Ganoderma applanatum* (*G. applanatum*) dispels wind to eliminate dampness and exhibited nephron- and liver-protective effects as noted in Chinese herbal classic literature; it might also affect hyperuricemia. Therefore, we examined the hypouricemia effects and mechanisms underlying *G. applanatum* on chemical-induced hyperuricemia in mice. Ethanol (GAE) and water (GAW) extracts were prepared by extracting *G. applanatum* in ethanol (GAE), followed by bathing the remains in water to yield GAW. GAE and GAW were administered orally at different doses to hyperuricemia mice, while allopurinol and benzbromarone served as positive controls. Both GAE and GAW showed remarkable hypouricemia activities, rendering a substantial decline in the SUA (serum uric acid) level in hyperuricemia control (*P* < 0.01). Moreover, the urine uric acid (UUA) levels were enhanced by GAE and GAW. In contrast to the evident renal toxicity of allopurinol, GAE and GAW did not show a distinct renal toxicity. Almost no suppressing effect was observed on the XOD activities. However, compared to the hyperuricemia control, OAT1 was elevated remarkably in mice drugged with GAE and GAW, while GLUT9 was significantly decreased. Similar to benzbromarone, GAE decreased the URAT1 protein levels significantly (*P* < 0.01), while GAW did not display a similar effect. GAE and GAW downregulated the level of CNT2 proteins in the gastrointestinal tract of hyperuricemia mice. Thus, *G. applanatum* produced outstanding hypouricemic effects, mediated by renal OAT1, GLUT9, and URAT1 and gastrointestinal CNT2 that might elevate urine uric secretions and decline in the absorption of purine in the gastrointestinal tracts. *G. applanatum* showed little negative influence on inner organs. By docking screening, four top-ranked compounds were identified that necessitated further investigation.

**Compounds:** potassium oxonate, hypoxanthine, allopurinol, benzbromarone.

## Introduction

Chronic diseases, suc as heart disease, diabetes, and gout are the leading factors of mortality worldwide, accounting for 60% of all deaths (Asaria et al., 2007). Among these, gout is characterized with reoccurring inflammatory arthritis, which is triggered by the crystallization of serum uric acid (SUA) in the joints and is often caused by hyperuricemia (Harris et al., 1999; Choi et al., 2005). Recently, several studies have been focused on seeking new agents from natural products for hyperuricemia and gout (Yu et al., 2007; Xu et al., 2013; Wu et al., 2014a) attributed to the soaring prevalence of hyperuricemia (Choi and Curhan, 2005; Liu et al., 2015) and the safety priority of natural products. Of these, traditional Chinese medicine (TCM) is frequently utilized for the prevention of hyperuricemia-related disorders (Wu et al., 2014b). In TCM, the medicinal fungi have been under intensive focus for several years (Wu et al., 2012; Xiao et al., 2012; Jiao et al., 2013; Ding et al., 2016; Yong et al., 2016; Chen D. et al., 2017); for example, many *Ganoderma* species are valued greatly (Paterson, 2006). *G. lucidum* is used for preventing chronic diseases, such as chronic hepatitis, cancer, arthritis, and nephritis (Chen et al., 2012). To date, more than 400 secondary metabolites have been isolated from *Ganoderma* species; polysaccharides and triterpenoids are the major bioactive metabolites of this genus. In 2016, our group reported a pair of structurally novel and anti-cancer compounds from *Ganoderma species* (Chen S. et al., 2017) with diverse pharmacological activities (Pan et al., 2015; Xie et al., 2016; Xiao et al., 2017). These compounds specifically exhibited nephron- and liver-protective effects as described previously (Shi et al., 2008; Zhou et al., 2010; Yan et al., 2015); kidney and liver are the major organs affecting hyperuricemia. Thus, we hypothesized that *G. applanatum* might be effective for hyperuricemia since it was recorded as a diuresis agent (Li, 2013) in Chinese herbal classic literature and closely associated with the prevention of hyperuricemia.

In this study, along with the on-going study of pharmacological activities of medicinal fungi (Yong et al., 2016; Chen S. et al., 2017), we firstly reported the hypouricemia effects of *G. applanatum* in hyperuricemia mice model established chemically. Thus, we prepared ethanol (GAE) and water (GAW) extracts by extracting *G. applanatum* with ethanol, followed by decocting the remains with water. The effects of GAE and GAW on SUA and UUA were examined in the conventional hyperuricemia models. In addition, BUN (blood urea nitrogen) and creatinine in serum and urine were assayed. Moreover, the influence of GAE and GAW on organ coefficients was also assessed to evaluate the effects on inner organs. In order to further elucidate the underlying mechanisms, the mRNA and protein levels of the key targets of hyperuricemia, including hepatic XOD together with renal GLUT9 (glucose transporter 9), UART1 (uric acid transporter 1), and OAT1 and gastrointestinal concentrative nucleoside transporter 2 (CNT2) were examined. To unveil the bioactive metabolites for hyperuricemia, virtual screening was performed using an in-house database for *G. applanatum.*

## Experimental

### Chemicals and Reagents

Hypoxanthine (HX, 98.5%), potassium oxonate (PO, 98%), benzbromarone (99%) and allopurinol (99.5%) were purchased from Aladdin Reagent Co. (Shanghai, China). Guangdong Yuewei Edible Fungi Technology Co. (Guangzhou, China) offered and confirmed the dried *G. applanatum* samples in May 2017. A voucher specimen (YW20170517-GA) was deposited in the herbarium of Guangdong Institute of Microbiology. SUA and XOD kits were obtained from Nanjing JianCheng Bioengineering Institute (Nanjing, China).

### Medicinal Fugal Extracts

*Ganoderma applanatum* (100 g) was immersed three times with 2 L of ethanol at 65°C for 3 h. Then, the extract was filtered and evaporated to yield GAE (3.78 g; 3.78%) and water extract (GAW; yield 8.53 g; 8.53%) was obtained by extracting the remains using water as solvent at 85°C, followed by lyophilization. The HPLC fingerprints (Supplementary Figures S1, S2) of GAE and GAW and standard chemical (ganoderic acid B, Supplementary Figure S3) were utilized as a future reference for identifying *G. applanatum*.

### Animals and Hyperuricemia Models

All experiments were approved and conducted by/in Guangdong Institute of Microbiology (GT-IACUC20170623-1; Guangzhou, China). Male Kunming mice (20 ± 2g) were obtained from Guangdong Provincial Medical Laboratory Animal Centre (Guangzhou, China). They were allowed to adapt to the experimental environment for 1 week. A previous protocol (Yong et al., 2016) was employed for modeling hyperuricemia. Primarily, all animals were randomized into normal, hyperuricemia, allopurinol, and benzbromarone controls (*n* = 10) and drug groups with GAE and GAW at low-, middle-, and high-doses (*n* = 10), wherein, PO (100 mg/kg) and HX (500 mg/kg) were injected for elevating SUA intraperitoneally and orally, respectively, simultaneously. Allopurinol and benzbromarone were used as positive controls.

### Drug Treatment

Primarily, mice were randomized (*n* = 10) into normal, hyperuricemia, allopurinol, and benzbromarone controls and groups treated by GAE and GAW of different doses. Normal and hyperuricemia controls were injected saline. Allopurinol (5 mg/kg) and benzbromarone (7.8 mg/kg) controls were treated with these drugs, respectively. GAE (30, 60, 120 mg/kg) and GAW (30, 60, 120 mg/kg were administered to drug groups, correspondingly. The drug administration continued for 1 week/day.

### Determination of SUA, BUN, Creatinine, and XOD

Serum and urine were collected for testing SUA (Carroll et al., 1971), BUN, and creatinine. Livers were homogenized for assaying the XOD activities according to the manufacturer’s instructions.

### Organ Coefficient

After the experiment, inner organs were collected and weighted. Specifically, organ coefficients were represented as the ratios of inner organs to the corresponding body weights.

### Statistical Analysis

Data were processed using SPSS (SPSS Inc.) by one-way analysis of variance (ANOVA) and expressed as mean ± standard error of mean (SEM). The difference was significant at *P* < 0.05 or *P* < 0.01 as compared by the two-tailed Student’s *t*-test.

### RT-PCR Analysis of *URAT1*, *GLUT9*, and *mOAT1*

Total RNA extractions were performed using TRIzol reagent. After homogenization of the kidney tissue, the obtained liquid was mixed with chloroform and centrifuged, followed by precipitating the aqueous phase with an equivalent volume of isopropanol. After washes with ethanol (75%), the total RNA pellets were suspended using DEPC water. The reverse transcription was conducted using 1 μg RNA with M-MLV reverse transcriptase. The obtained cDNA was diluted with DNase-free water and PCR amplification was performed using primers at appropriate conditions (**Table 1**); *GAPDH* was used as an endogenous standard. Finally, the PCR products were quantified by electrophoresis.

**Table 1:** PCR primer sequences and protocols.

Description	GenBank	Sense primer (5′–3′)	Antisense primer (5′–3′)	Product size (bp)	T_m_ (°C)	Thermal cycle
GAPDH	NM_008084.2	GTTCCTACCCCCAATGTGTCC	TAGCCCAAGATGCCCTTCAGT	125	60	40
GLUT9	NM_001012363.2	GATGCTCATTGTGGGACGGTT	CTGGACCAAGGCAGGGACAA	241	60	40
URAT1	NM_009203.3	CGCTTCCGACAACCTCAATG	CTTCTGCGCCCAAACCTATCT	254	60	40
OAT1	NM_008766.3	GCCTTGATGGCTGGGTCTATG	AGCCAAAGACATGCCCGAGA	287	60	40


### Western Blot

After three PBS washes, the kidney cortexes were homogenized with 10 equivalent volumes of RIPA lysis buffer, supplemented with 1 mM PMSF (protease inhibitor), in an ice bath. Following incubation on ice for 30 min, the mixtures were centrifuged (12000 × *g*, 10 min), and the supernatants were the total proteins; the concentration was determined by BCA Protein Assay Kit (Tiangen Biotech Co., China). An equivalent of 5 μg protein samples were separated by 10% SDS-PAGE and transferred to PVDF membrane (Millipore, United States). The non-specific binding sites of the membranes were blocked with 5% skimmed milk in TBST (Tris-buffered saline with 0.1% Tween-20) mixed. Then, the membranes were probed overnight with specific primary antibodies (**Table 2**) diluted in TBST: URAT1 (1:2000), GLUT9 (1:2000), OAT1 (1:2000), and GAPDH (1:4000), followed by secondary HRP-conjugated goat anti-rabbit IgG (1:3000) antibody for 30 min. Consequently, the membranes were mixed with ECL (Enhanced Chemiluminescence, Servicebio Co., China) and exposed to X-ray film. The immunoreactivity of the target proteins was analyzed via densitometry using Alpha Innotech (AlphaEase Shop, United States) and normalized against that of GAPDH.

**Table 2:** Antibodies for Western blotting analysis.

Company	Description	Catalog number
ProteinTech Group (Chicago, IL, United States)	Rabbit URAT1 Antibody	14937-1-AP
Novus Biologicals (Littleton, CO, United States)	Rabbit GLUT9 Antibody	NBP1-05054
Abcam Inc. (Cambridge, MA, United States)	OAT1 Antibody	ab135924
Bioworld Technology Inc. (St. Louis Park, MN, United States)	CNT2 Antibody	BS5670
Servicebio Co. (Wuhan, China)	GAPDH antibody	GB13002-m-1


### OAT1 Modeling and Docking Screening

OAT1 modeling was performed using Modeler (Webb and Sali, 2014) with EasyModeller 4.0 (Kuntal et al., 2010) as the graphical interface. The OAT1 sequence was obtained from UniProt (ID: Q4U2R8-1), and 4JA3 and 4PYP served as the templates. An in-house compound database for *G. applanatum* was established based on the literature on the compounds of *G. applanatum*. Then, docking was conducted with Gold (Adeniyi and Ajibade, 2013) and binding energy calculated by the MM-GBSA method.

## Results

**FIGURE 1:**
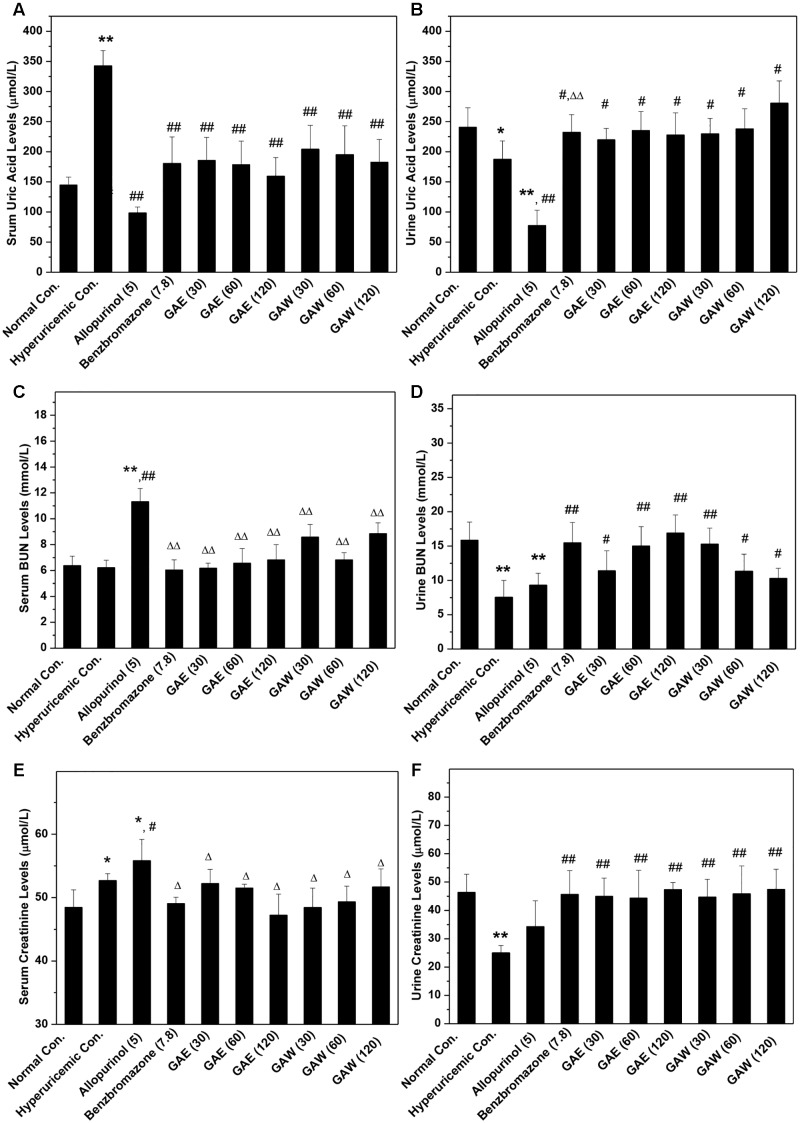
Effects of *G. applanatum* on serum uric acid **(A)**, BUN **(B)**, and creatinine **(C)** and urine uric acid **(D)**, BUN **(E)**, and creatinine **(F)** in mice. ^∗^*P* < 0.05, ^∗∗^*P* < 0.01 compared to the normal control; ^#^*P* < 0.05, ^##^*P* < 0.01 as compared to the hyperuricemia control; ^Δ^*P* < 0.05, ^ΔΔ^*P* < 0.01 compared to the allopurinol control.

The model was established successfully, which was further confirmed by the remarkable increase in SUA up to 342 μmol/L in the hyperuricemia control (*P* < 0.01) as compared to that of normal mice (145 μmol/L, **Figure 1A**); also a decline in SUA in allopurinol (98 μmol/L, *P* < 0.01) and benzbromarone (181 μmol/L, *P* < 0.01) controls was noted. Importantly, GAE at 30, 60, and 120 mg/kg doses were ascribed to the decrease in the level of SUA to 185, 178, and 159 μmol/L (*P* < 0.01) in hyperuricemia mice. Moreover, the doses of GAW at 30, 60, and 120 mg/kg led to the decrease in SUA to 204, 195, and 182 μmol/L (*P* < 0.01). Both GAE and GAW exhibited substantial hypouricemia activities in this model.

In order to further explore the effects of GAE and GAW, the levels of UUA were assayed (**Figure 1B**). The reduction in UUA was observed in the model control (187 μmol/L) as compared to that of the normal mice (241 μmol/L, *P* < 0.05), which was further decreased by allopurinol dose at 78 μmol/L (*P* < 0.01). This decline was restored by the administration of benzbromarone at 7.8 mg/kg, GAE at 30, 60, and 120 mg/kg and GAW at 30, 60, and 120 mg/kg to 232, 220, 235, 227, 229, 238, and 280 μmol/L (*P* < 0.05), correspondingly.

To assess the changes in the renal function, the parameters were assayed in hyperuricemia mice. Since only a small dose of PO was required for establishing the model, the increase in serum BUN levels in hyperuricemia mice (6.2 mmol/L) by administering PO in normal mice (6.3 mmol/L, *P* > 0.05) was not observed, which was consistent with the previous reports (**Figure 1C**). Allopurinol (11.3 mmol/L, *P* < 0.01) elevated the serum BUN, indicating an impairment of renal function. However, GAE and GAW exhibit serum BUN parameters of 6.2, 6.5, and 6.8 mmol/L for GAE at different doses and 8.5, 6.8, and 8.8 mmol/L for GAW at various doses, respectively (*P* < 0.01), much lower than that of the allopurinol control.

Administration of PO and HX rendered a decrease in urine BUN levels (7.55 mmol/L) in normal mice (15.86 mmol/L, *P* < 0.01, **Figure 1D**). Benzbromarone (15.48 mmol/L, *P* < 0.01) increased the level of urine BUN. In comparison to the hyperuricemia control, urine BUN was elevated significantly by GAE and GAW to 11.40, 15.02, and 16.91 mmol/L and 15.27, 11.35, and 10.30 mmol/L (*P* < 0.01) at the doses of 30, 60, and 120 mg/kg and 30, 60, and 120 mg/kg correspondingly, showing an elevated handling effects of renal organ.

The hyperuricemia control (52.7 μmol/L) elevated the serum creatinine as compared to the normal control (48.5 μmol/L, *P* < 0.05, **Figure 1E**). Allopurinol (55.8 μmol/L; *P* < 0.01 as compared to the normal control, *P* < 0.05 as compared to the hyperuricemia control) increased the serum creatinine, demonstrating damage to the renal function. However, GAE and GAW with various doses presented serum creatinine at 52.2, 51.5, and 47.2 and 48.4, 49.3, and 51.7 μmol/L, respectively, similar to the normal control. Notably, GAE and GAW may not affect the renal function negatively, which was similar to that with BUN analysis.

In comparison to the normal control (46.36 μmol/L), PO and HX decreased the urine creatinine (25.04 μmol/L, *P* < 0.01), and allopurinol (34.27 μmol/L, *P* < 0.01) also showed a similar effect (**Figure 1F**). Apparently, GAE and GAW significantly increased the creatinine levels to 45.00, 44.35, and 47.37 and 44.70, 45.8,5 and 47.45 μmol/L (*P* < 0.01), respectively, at the corresponding doses in hyperuricemia mice.

**FIGURE 2:**
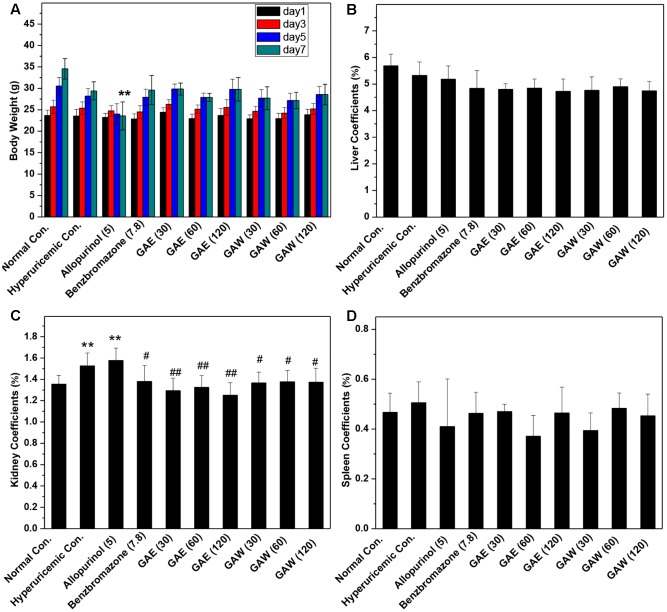
Effects of *G. applanatum* extracts on **(A)** body weight, **(B)** liver coefficients, **(C)** kidney coefficients, and **(D)** spleen coefficients in mice. ^∗∗^*P* < 0.01 as compared to the normal control; ^#^*P* < 0.05, ^##^*P* < 0.01 as compared to the hyperuricemia control.

In comparison to the normal control, allopurinol suppressed the natural growth of body weights, significantly (*P* < 0.01, **Figure 2A**). However, GAE and GAW did not affect the increase in body weight. The liver coefficients for all groups were not different significantly, demonstrating little negative effect of GAE and GAW on liver function (**Figure 2B**). Hyperuricemia (1.52%) and allopurinol (1.57%) regulate the kidney coefficients that were enhanced in comparison to the normal control (1.35%, *P* < 0.01), indicating negative influences of PO and allopurinol (**Figure 2C**). GAE and GAW exhibited the indicators of 1.38, 1.30, and 1.32% and 1.36%, 1.37%, and 1.37% without any difference as compared to the normal control (*P* > 0.05). Thus, no negative influences of GAE and GAW were observed, which was in line with BUN and creatinine analysis. Moreover, no significant difference was observed between the spleen coefficients (**Figure 2D**).

**FIGURE 3:**
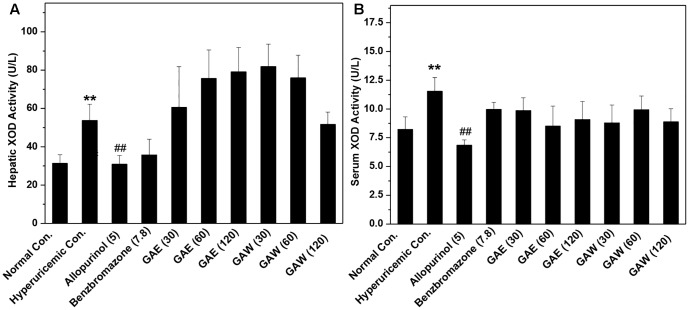
Effects of *G. applanatum* extracts on XOD activities in the liver **(A)** and serum **(B)** in mice. ^∗∗^*P* < 0.01 as compared to the normal control; ^##^*P* < 0.01 as compared to the hyperuricemia control.

Compared to the hyperuricemia control, allopurinol lowered the hepatic and serum XOD activities (*P* < 0.01, **Figure 3**). However, GAE and GAW did not demonstrate any suppressive effects on XOD. Therefore, hypouricemic effects of GAE and GAW might not be due to the inhibitory effects of XOD activities, but rather due to the uricosuria effects in hyperuricemia mice according to the UUA analysis.

**FIGURE 4:**
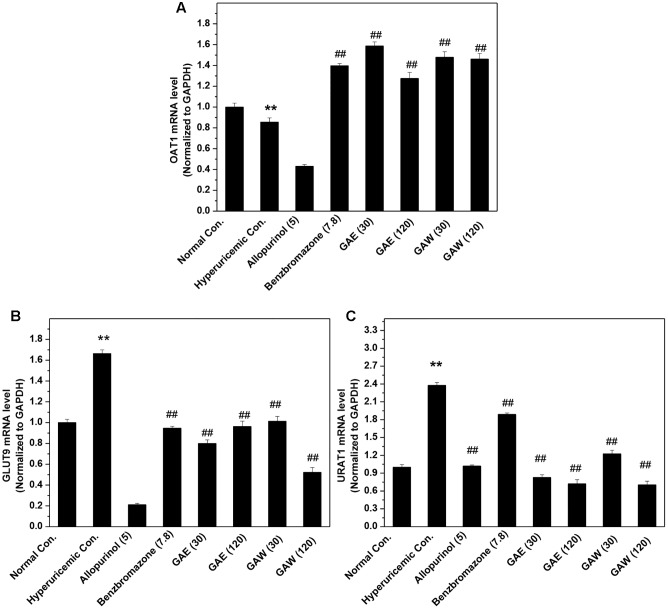
Effects of GAE and GAW for renal **(A)** OAT1, **(B)** GLUT9, and **(C)** URAT1 mRNA. ^∗∗^*P* < 0.01 compared to the normal control; ^##^*P* < 0.01 as compared to the hyperuricemia control.

The influences of GAE and GAW on renal transporter mRNA were illustrated in **Figure 4**. The OAT1 mRNA level was reduced significantly by PO and HX in hyperuricemia control (*P* < 0.01) *vs*. normal control (**Figure 4A**). Similar to benzbromarone, GAE, and GAW (*P* < 0.01) were enhance the OAT1 level as compared to the hyperuricemia control. In addition, GAE and GAW (*P* < 0.01) significantly reversed the elevation of *GLUT9* mRNA in hyperuricemia mice (**Figure 4B**). Allopurinol and benzbromarone were found to decrease the level of *GLUT9* mRNA in hyperuricemia models. PO and HX utilized for hyperuricemia model were inclined to enhance the *URAT1* mRNA as compared to the normal control (**Figure 4C**). GAE and GAW (*P* < 0.01) downregulated the level of *URAT1* mRNA *vs*. hyperuricemia control; allopurinol and benzbromarone also exhibited a similar effect.

**FIGURE 5:**
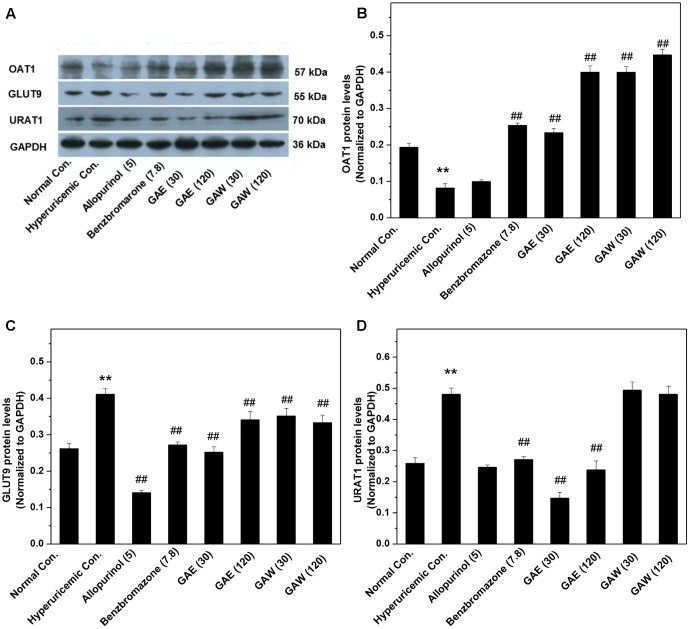
Effects of *G. applanatum* extracts on the protein levels of renal ion transporters. The kidney cortex protein was extracted for Western blot analysis of OAT1 **(A,B)**, GLUT9 **(A,C)**, and URAT1 **(A,D)**. ^∗∗^*P* < 0.01 as compared to the normal control; ^##^*P* < 0.01 as compared to the hyperuricemia control.

Additionally, the impacts of *G. applanatum* on renal OAT1, GLUT9, and URAT1 proteins were examined by Western blotting (**Figure 5**). Similar to benzbromarone, GAE and GAW increased the level of OAT1 remarkably as compared to the hyperuricemia control (*P* < 0.01). Moreover, the expression of GLUT9 was downregulated (*P* < 0.01) significantly. GAE decreased the level of URAT1 protein significantly (*P* < 0.01), while GAW did not display a similar effect. Reportedly, the regulation of the above targets was correlated to the UUA elevation.

**FIGURE 6:**
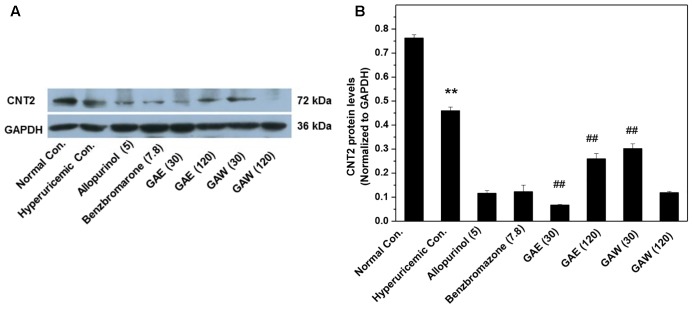
Effects of *G. applanatum* extracts on protein levels of intestines transporters. Kidney cortex protein was extracted for Western blot analysis of CNT2 **(A,B)**. ^∗∗^*P* < 0.01 as compared to the normal control; ^##^*P* < 0.01 as compared to the hyperuricemia control.

Furthermore, the effects of *G. applanatum* on the gastrointestinal CNT2 protein levels were examined (**Figure 6**). GAE and GAW downregulated the CNT2 protein levels in the intestine of hyperuricemia mice (*P* < 0.01).

**FIGURE 7:**
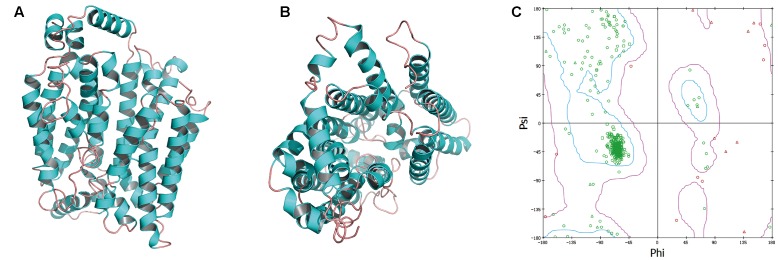
The side **(A)** and cytoplasmic **(B)** views of modeled OAT1 structure and its Ramachandran map **(C)**.

GAE and GAW regulated the level of OAT1, and thus, we constructed the OAT1 structure through homology modeling owing to the absence of crystallographic data. Based on sequence alignments, 4JA3 and 4PYP with high identities > 30% and low *E*-values were chosen as templates for the establishment of the model. The optimal model (**Figures 7A,B**) was selected and then validated through the Ramachandran plot (**Figure 7C**), identifying 92.6% as the most favored residues, and the allowed were 5.9%. These findings supported the high quality of this model that was exploited for virtual screening.

**FIGURE 8:**
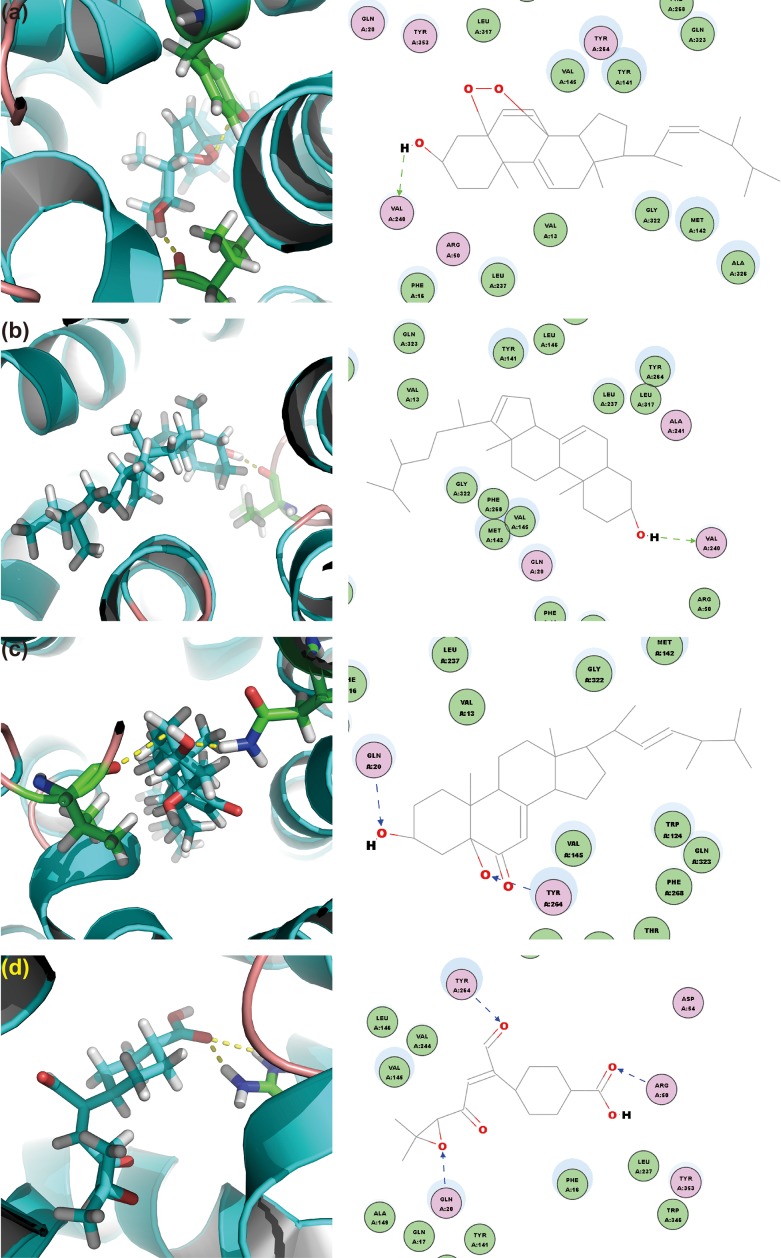
OAT1 with top-ranked four ligands of *G. applanatum*: **(a)** OAT1-384; **(b)** OAT1-410; **(c)** OAT1-386; **(d)** OAT1-377. Green and blue lines represent the hydrogen bonds.

To screen the putative bioactives for hyperuricemia from *G. applanatum*, we docked the compounds of our in-house database of *G. applanatum* to OAT1. Among these docked compounds, compound 384, 410, 386, and 377 were ranked highly. Of these, compound 384 had the binding energy of -80.09 kcal/mol (**Figure 8a**). The residue VAL240 formed a hydrogen bond with the hydrogen atom of the hydroxyl attached to the four-cyclic moiety. The compound 410 showed the binding energy of -86.25 kcal/mol (**Figure 8b**). On the other hand, VAL240 formed a hydrogen bond with the hydrogen atom of the hydroxyl group attached to the four-cyclic moiety. The compound 386 exhibited a binding energy of -129.89 kcal/mol (**Figure 8c**); of these, the hydrogen atoms of hydroxyls attached to the four-cyclic moiety formed two hydrogen bonds with GLN20 and TYR264, respectively. The compound 377 demonstrated a binding energy of -140.35 kcal/mol (**Figure 8d**). Herein, TYR264 and ARG50 offered two hydrogen bonds to the two carbonyl groups attached to the cyclohexane moiety. GLN20 formed a hydrogen bond with the epoxy group on the side chain.

## Discussion

Herein, we investigated the hypouricemia activities of *G. applanatum*, utilizing the ethanol and water extracts in hyperuricemia models established by PO and HX. The pharmacological activity for hyperuricemia was correlated to the regulation of key targets for hyperuricemia; for example, renal OAT1, GLUT9, and URAT1 and gastrointestinal CNT2.

PO and HX are frequently employed to establish the animal models for medical investigations of hyperuricemia. In the present study, PO and HX together induced hyperuricemia, which was further confirmed by a dramatic increase in the SUA level (Yong et al., 2016). In this model, the hypouricemia effects of *G. applanatum* extracts were investigated. GAE and GAW remarkably lowered the level of SUA via elevated UUA levels in hyperuricemia mice, thereby confirming the uricosuria effect of *G. applanatum* experimentally, as described previously (Li, 2013).

Creatinine and BUN are critical indicators for renal function, as its impairment is usually accompanied by an increase in the level of the two indicators in serum and decrease in the urine. Owing to the small usage of PO, the impairment of kidney by PO in hyperuricemia mice was not observed in this model, which was consistent with the pathology of asymptomatic hyperuricemia reported previously. In contrast to the evident renal toxicity of allopurinol (Horiuchi et al., 2000), the administration of GAE and GAW did not elicit renal toxicity.

To elucidate the mechanisms underlying the hypouricemia action of *G. applanatum*, we tested its effects on the XOD activities (Cao et al., 2011). GAE and GAW did not decrease the XOD activity, demonstrating that the hypouricemia effects of *G. applanatum* may be raised by other methods, such as uricosuria or anti-purine absorption pathways. This phenomenon was consistent with the elevated levels of UUA. Clinically, approximately 90% of the hyperuricemia patients had a raised level of the under-excretion of renal uric acid (Ichida et al., 2012). Renal transporters, including OAT1, GLUT9, and URAT1, are directly associated with the homeostasis of SUA (Enomoto et al., 2002; Anzai et al., 2010). Among them, OAT1 plays a significant role in renal uric acid excretion initially (Nigam et al., 2007). GAE and GAW increased the mRNA and protein levels of OAT1, inducing the remarkably enhanced uric acid excretion by advancing the uric acid transportation through OAT1. PO and HX were used in the model for upregulation of the GLUT9 mRNA and protein levels (Doring et al., 2008). These were alleviated by GAE and GAW, thereby enhancing the excretion of uric acid by inhibiting the uric acid reabsorption through GLUT9. Furthermore, GAE downregulated the level of URAT1 protein (Enomoto et al., 2002); however, GAW did not show this effect. Since URAT1 functions for the reabsorption of uric acid through the kidney, GAW may elevate the uric acid excretion. Notably, the upregulation of OAT1 and downregulation of GLUT9 and URAT1 correlated to the increase in UUA in GAE- and GAW-treated groups. These findings provided the evidence that the synergistic activity of OAT1, GLUT9, and URAT1 by *G. applanatum* contributed to the uricosuria actions of *G. applanatum* in hyperuricemia mice. Moreover, the gastrointestinal level of CNT2 (Johnson et al., 2012) is a purine nucleoside-preferring transporter playing a substantial role in purine absorption in the enterocyte from food. Herein, both GAE and GAW severely downregulated the CNT2 protein levels, demonstrating the inhibitory effects on the absorption of purines in the gastrointestinal tract. Thus, all the above synergistic effects of purine absorption inhibition and uricosuria actions might be attributed to the hypouricemic effects of *G. applanatum*. Owing to the excellent pharmacological activity of *G. applanatum*, a further study of screening the bioactive metabolites of *G. applanatum* is essential for the above targets by computational and experimental techniques together.

In order to predict the bioactives in *G. applanatum* for hyperuricemia, molecular docking virtual screening was performed. Primarily, a high-quality OAT1 structure was established by homology modeling using 4JA3 and 4PYP as templates, the favored and allowed residues account for 98.5%. Subsequently, a library of compounds collected in literature for *G. applanatum* was built by sketching and energy minimization. With the library and the modeled OAT1 structure, docking was conducted, and four top-ranking compounds were selected for a detailed analysis. Consequently, the four compounds had a binding energy between -80 and -150 kcal/mol and hydrogen bonds were involved in the orientations and interactions of each compound, necessitating further studies.

In summary, we reported that the hypouricemia effects of *G. applanatum* extract in hyperuricemia mice were attributed to the regulation of renal OAT1, GLUT9, and URAT1 and gastrointestinal CNT2 for GAE and OAT1 and GLUT9 and gastrointestinal CNT2 for GAW. Therefore, the synergistic effects of purine absorption inhibition and uricosuria activities may be attributed to the hypouricemia effects of *G. applanatum*. To identify the bioactives for hyperuricemia in *G. applanatum*, computational screening was performed with modeled OAT1 structure and collected compounds for *G. applanatum* in literature and four top-ranked compounds were analyzed detail, with the binding energy between -80 and -150 kcal/mol; the hydrogen bonds were involved in orientations and interactions for each compound.

## Ethics Statement

This study was carried out in accordance with the recommendations of Animal Experiment Guidlines, Committee of Guangdong Institute of Microbiology. The protocol was approved by the Committee of Guangdong Institute of Microbiology.

## Author Contributions

TY conceived and designed the study. The experiments were performed by TY. The manuscript was written by TY and approved by all authors. SC, YX, DC, JS, OS, CJ, and DZ helped in the experiments.

## Conflict of Interest Statement

OS and CJ was employed by Guangdong Yuewei Edible Fungi Technology Co. The other authors declare that the research was conducted in the absence of any commercial or financial relationships that could be construed as a potential conflict of interest.

## Abbreviations

BUN: blood urine nitrogen
CNT2: gastrointestinal concentrative nucleoside transporter 2
GAE: ethanol extract of *G. applanatum*
GAW: water extract of *G. applanatum*
GLUT9: glucose transporter 9
HX: hypoxanthine
OAT1: organic anion transporter 1
PO: potassium oxonate
SUA: serum uric acid
UART1: uric acid transporter 1
UUA: urine uric acid
XOD: xanthine oxidase
