# Supplementary material for: Hypouricemic Effects of Ganoderma applanatum in Hyperuricemia Mice through OAT1 and GLUT9
Source: Front Pharmacol. 2018 Jan 15;8:996. doi: 10.3389/fphar.2017.00996 (PMC5775298; doi:10.3389/fphar.2017.00996)
Supplement: Supplementary file 1 [file Table_1.DOC]

**Submission to Frontiers in Pharmacology**

Supporting Information

**Hypouricemic Effects of Extracts from Ganoderma applanatum in Hyperuricemic Mice through OAT1 and GLUT9**

Tianqiao Yong ^a, b^, *, Shaodan Chen ^a, b^, Yizhen Xie ^a,b^, Diling Chen ^a^, Jiyan Su ^a^, Ou Shuai ^a^, Chunwei Jiao ^b^, Dan Zuo ^c^

^a^ State Key Laboratory of Applied Microbiology Southern China, Guangdong Provincial Key Laboratory of Microbial Culture Collection and Application and Guangdong Open Laboratory of Applied Microbiology, Guangdong Institute of Microbiology, Guangzhou 510070, China

^b^ Guangdong Yuewei Edible Fungi Technology Co., Guangzhou 510663, China

^c^ Guangzhou Institutes of Biomedicine and Health, Chinese Academy of Sciences, Guangzhou 510530, China

***Corresponding author:**

Tianqiao Yong, Fax: +86-20-32059602, E-mail: tianqiao@mail.ustc.edu.cn

**The following is included as additional supporting materials for this paper:**

Page S4 **Fig. S1** The fingerprint of GAE. HPLC conditions-column: Waters Atlantis T3 RP-C18 column, 5μm, 250 mm × 4.6 mm; the mobile phases: acetonitrile (28-100% in 115 min) and 0.01 % acetic acid aqueous solution (72-0% in 115 min), flowing rate: 1 ml/min; detection wavelength: 250 nm; temperature: 25 ^o^C; injection: 20 μL.

Page S4 **Fig. S2** The fingerprint of GAW. HPLC conditions-column: Waters Atlantis T3 RP-C18 column, 5μm, 250 mm × 4.6 mm; the mobile phases: acetonitrile (28-100% in 115 min) and 0.01 % acetic acid aqueous solution (72-0% in 115 min), flowing rate: 1 ml/min; detection wavelength: 250 nm; temperature: 25 ^o^C; injection: 20 μL.

Page S4 **Fig. S3** A HPLC chromatogram of the standard chemicals (Ganoderic acid B) for *G. applanatum* identification. HPLC conditions-column: Waters Atlantis T3 RP-C18 column, 5μm, 250 mm × 4.6 mm; the mobile phases: acetonitrile (28-100% in 115 min) and 0.01 % acetic acid aqueous solution (72-0% in 115 min), flowing rate: 1 ml/min; detection wavelength: 250 nm; temperature: 25 ^o^C; injection: 20 μL.

**Experimental**

1. *HPLC conditions*

Chromatography was performed on a reversed-phase column (Waters Atlantis T3 RP-C18 column, 5μm, 250 mm × 4.6 mm) with a flow rate of 1 ml/min. Separation was carried out by programed gradient elution with acetonitrile (28-100% in 115 min) and 0.01 % acetic acid aqueous solution (72-0% in 115 min). The detection wavelength was set at 250 nm, and the column temperature was kept at 25 ^o^C. The loading volume was 20 μl.

**Fig. S1**

**Fig. S2**

**Fig. S3**
